# Social Epidemiology of Dual Use of Electronic and Combustible Cigarettes Among U.S. Adults: Insights from the Population Assessment of Tobacco and Health (PATH) Study

**DOI:** 10.31586/gjcd.2024.1131

**Published:** 2024-11-19

**Authors:** Shervin Assari, Payam Sheikhattari

**Affiliations:** 1 Department of Internal Medicine, Charles R Drew University of Medicine and Science, Los Angeles, CA, USA; 2 Department of Urban Public Health, Charles R Drew University of Medicine and Science, Los Angeles, CA, USA; 3 Center for Urban Health Disparities Research and Innovation, Morgan State University, Baltimore, MD, USA; 4 The Prevention Sciences Research Center, School of Community Health and Policy, Morgan State University, Baltimore, MD, USA; 5 Department of Public and Allied Health, School of Community Health and Policy, Morgan State University, Baltimore, MD, USA

**Keywords:** Dual Use, E-Cigarettes, Combustible Cigarettes, Predictors, Sociodemographic Factors

## Abstract

**Background:**

The dual use of e-cigarettes and combustible cigarettes poses significant public health concerns due to the compounded risks associated with the use of both products. Understanding the predictors of dual use can inform targeted interventions and tobacco control strategies aimed at reducing nicotine dependence and health risks among adults.

**Objective:**

This study aims to identify the sociodemographic predictors of dual use of e-cigarettes and combustible cigarettes among U.S. adults using baseline data from the Population Assessment of Tobacco and Health (PATH) Study.

**Methods:**

We analyzed baseline data from the PATH Study, focusing on adult participants who reported the use of both e-cigarettes and combustible cigarettes. Logistic regression models were used to identify the associations between dual use and key sociodemographic variables, including age, gender, race/ethnicity, and education level.

**Results:**

The analysis revealed that dual use of e-cigarettes and combustible cigarettes was predominantly observed among young, female, non-Latino, White, and highly educated adults. Younger adults were more likely to engage in dual use compared to older age groups. Females showed higher rates of dual use compared to males. Non-Latino White individuals were more likely to be dual users than individuals from other racial/ethnic backgrounds. Additionally, higher educational attainment was associated with increased dual use, contrary to traditional smoking patterns.

**Conclusion:**

The findings highlight specific demographic groups that are at higher risk of dual use of e-cigarettes and combustible cigarettes, particularly younger, highly educated, non-Latino White females. These insights suggest the need for tailored public health interventions that address the unique needs and behaviors of these populations. Future research should explore the underlying motivations and contextual factors contributing to dual use to enhance the effectiveness of tobacco control policies and cessation programs.

## 1. Introduction

The rise of electronic cigarettes (e-cigarettes) has introduced a new landscape in tobacco use, with a growing prevalence of dual use of e-cigarettes and combustible cigarettes among adults in the United States [1–3]. E-cigarettes have gained popularity as alternatives to traditional smoking, particularly among individuals seeking to reduce or quit smoking [1,4]. These products are often perceived as less harmful due to the absence of combustion, which reduces exposure to many of the toxicants found in cigarette smoke [5,6]. However, the dual use of e-cigarettes alongside combustible cigarettes raises significant public health concerns [7–10]. While e-cigarettes may reduce some of the harms associated with smoking, the concurrent use of both products sustains nicotine dependence and prolongs exposure to the toxic and carcinogenic substances found in traditional cigarettes, negating many potential benefits of switching to e-cigarettes alone [7–10].

Dual use is particularly problematic because it complicates efforts to quit smoking entirely and may lead to increased overall nicotine intake and dependence [7–10]. This behavior can also perpetuate the social acceptability of smoking-like behaviors and delay full cessation, undermining public health goals to reduce smoking prevalence and related diseases. Furthermore, the dual use of e-cigarettes and combustible cigarettes may result in unique patterns of exposure to various harmful constituents, which could exacerbate the risk of adverse health outcomes such as cardiovascular disease, respiratory problems, and cancer [7–10]. As the landscape of tobacco use evolves with the growing presence of e-cigarettes, understanding the dynamics of dual use becomes increasingly important for guiding public health interventions and regulatory actions.

While e-cigarettes are often marketed as safer alternatives to traditional cigarettes and potential cessation aids, dual users continue to face significant health risks due to continued exposure to harmful chemicals in combustible cigarettes [3,5,11–15]. This dual use undermines the potential harm reduction benefits of e-cigarettes and poses a challenge for tobacco control efforts aimed at reducing the health burden of smoking. Moreover, dual use may not only perpetuate individual health risks but also contribute to broader public health issues by sustaining the tobacco market and complicating smoking cessation trends at the population level.

Understanding the demographic, behavioral, and psychosocial predictors of dual use is crucial for informing targeted interventions and public health strategies aimed at reducing tobacco-related harm. Identifying which groups are most likely to engage in dual use can help tailor interventions and messaging to address the specific needs and behaviors of these populations. Previous studies have indicated that dual use is more common among certain demographic groups, including younger adults, who are often more susceptible to novel tobacco products due to their higher likelihood of experimentation and sensation-seeking behaviors. Additionally, factors such as gender, race/ethnicity, and socioeconomic status have been linked to patterns of tobacco use, highlighting the complex interplay of individual and contextual factors that influence dual use.

However, the specific predictors of dual use in the adult population remain underexplored, particularly in the context of diverse sociodemographic backgrounds. As the tobacco product landscape continues to diversify, there is a pressing need to better understand how sociodemographic characteristics such as age, gender, race/ethnicity, and education level interact with dual use behaviors. In the current study, we aim to investigate the predictors of dual use of e-cigarettes and combustible cigarettes among U.S. adults using baseline data from the Population Assessment of Tobacco and Health (PATH) Study. Specifically, we will examine the sociodemographic characteristics associated with an increased likelihood of dual use. Our findings will contribute to a better understanding of the factors driving dual use in this population and support the development of targeted interventions to address this high-risk behavior, ultimately informing policies and practices that can effectively reduce tobacco-related morbidity and mortality.

## 2. Methods

### 2.1. Study Design

This study employed cross-sectional data from the Population Assessment of Tobacco and Health (PATH) Study [16,17] wave 1. The PATH Study is a nationally representative cohort study aimed at assessing tobacco use and its health impacts across a diverse adult population in the United States. For this analysis, we included participants who were adults and current smokers at baseline (Wave 1).

### 2.2. Participants

The study focused on individuals who were current smokers at baseline. Inclusion criteria required participants to be aged 18 years or older at Wave 1.

### 2.3. Variables and Measures

The primary outcome was use of current use of e-cigarettes in current smokers, defined as dual use at baseline. Smoking status could be daily or someday but was current. Similarly, e-cig use could be daily or some days a week but should be current smokers (smoked cigarettes at least a few days in the past week).

Independent variables included demographic factors such as age, sex, race/ethnicity, and educational attainment, poverty rate (living user the federal poverty line), and health insurance (Some private insurance, Private insurance, some Medicare Private insurance or Medicare, Other insurance only, and No insurance).

### 2.4. Statistical Analysis

Descriptive statistics were calculated to summarize the characteristics of the study sample, using proportions (weighted) for categorical variables. We conducted logistic regression analyses using Stata to explore predictors of dual use in current smokers at wave 1. Logistic regression models were performed with age, sex, race/ethnicity, and educational attainment, poverty rate, and health insurance as categorical predictors. All statistical analyses were performed using Stata, with a significance level set at p < 0.05 for all tests.

We used survey design variables, including weights, strata, and primary sampling units (PSUs), in conjunction with the subpop option to focus specifically on daily smokers at baseline. This approach ensures that our findings are representative of the U.S. daily smoker population by accounting for the complex sampling design of the Population Assessment of Tobacco and Health (PATH) Study. By using these design variables, we adjusted for differential probabilities of selection, non-response, and post-stratification adjustments, thereby improving the generalizability of the results to the broader U.S. population of daily smokers. This methodology allows us to accurately reflect national estimates of smoking cessation behaviors and related factors among U.S. adults who were daily smokers at the study’s outset.

### 2.5. Ethical Considerations

The PATH Study received institutional review board approval from the coordinating centers, and all participants provided informed consent. Data used in this analysis were de-identified to maintain participant confidentiality.

## 3. Results

Table 1 presents the descriptive characteristics of current smokers, with data weighted to reflect the U.S. adult population. The age distribution among daily smokers showed that the largest group was adults aged 25 to 34 years, accounting for 24.1% (SE = 0.005) of the sample, followed by those aged 45 to 54 years at 19.5% (SE = 0.004) and those aged 35 to 44 years at 18.9% (SE = 0.004). The youngest age group, 18 to 24 years, comprised 15.5% (SE = 0.004), while older adults were less represented, with those aged 65 to 74 years and 75 years or older making up 5.5% (SE = 0.003) and 1.5% (SE = 0.001) of the sample, respectively.

Regarding sex, the distribution was skewed slightly towards males, who comprised 55.8% (SE = 0.005) of daily smokers, while females made up 44.2% (SE = 0.005). In terms of ethnicity, the vast majority of daily smokers were non-Hispanic (86.7%, SE = 0.009), with Hispanic individuals accounting for 13.3% (SE = 0.009). When examining race, White individuals represented the majority of daily smokers at 75.1% (SE = 0.014), followed by Black individuals at 16.3% (SE = 0.013), and those identifying as other races at 8.6% (SE = 0.005).

Geographically, daily smokers were most prevalent in the South (40.5%, SE = 0.013), followed by the Midwest (24.0%, SE = 0.013), the West (18.9%, SE = 0.010), and the Northeast (16.5%, SE = 0.012). Educational attainment among daily smokers varied, with the highest representation among those with some college education or an associate degree (32.8%, SE = 0.005), followed by high school graduates (28.5%, SE = 0.006). Those with less than a high school education made up 16.8% (SE = 0.005), and individuals with a GED accounted for 10.7% (SE = 0.003). Smokers with a bachelor’s degree comprised 8.6% (SE = 0.004), while those with an advanced degree were the least represented at 2.6% (SE = 0.002).

In terms of poverty status, 39.6% (SE = 0.007) of daily smokers were living in poverty, while 60.4% (SE = 0.007) were not. Health insurance coverage among daily smokers showed that about half had some form of private insurance (49.1%, SE = 0.007). A smaller proportion had private insurance combined with some Medicare coverage (8.9%, SE = 0.004), or private insurance or Medicare with some other type (13.0%, SE = 0.005). Those with other insurance only made up 3.4% (SE = 0.002), and 25.6% (SE = 0.005) of daily smokers had no insurance.

Lastly, the prevalence of dual use among daily smokers was notable, with 20.8% (SE = 0.005) of daily smokers engaging in both e-cigarette and combustible cigarette use, while the majority, 79.2% (SE = 0.005), did not report dual use.

Table 2 presents the predictors of dual use of e-cigarettes and combustible cigarettes among current smokers (defined as those who smoke daily or on some days at baseline). The odds ratios (OR) indicate the likelihood of dual use in relation to the reference categories within each demographic variable. Age was a significant predictor of dual use, with younger adults showing higher odds compared to older age groups. Specifically, compared to the reference group (18 to 24 years old), adults aged 25 to 34 years had a slightly lower likelihood of dual use (OR = 0.853, 95% CI: 0.736–0.989, p = 0.035). The likelihood of dual use continued to decrease significantly with increasing age, with the lowest odds observed among adults 75 years or older (OR = 0.091, 95% CI: 0.041–0.203, p < 0.001). Males were less likely to engage in dual use compared to females, with an odds ratio of 0.894 (95% CI: 0.814–0.982, p = 0.019), suggesting a slightly lower prevalence of dual use among men. Hispanic ethnicity was associated with a lower likelihood of dual use compared to non-Hispanics (OR = 0.849, 95% CI: 0.738–0.976, p = 0.022). Race significantly influenced dual use, with Black adults having significantly lower odds of dual use compared to White adults (OR = 0.565, 95% CI: 0.471–0.677, p < 0.001). There was no significant difference in dual use between White adults and those from other races (OR = 1.041, 95% CI: 0.874–1.239, p = 0.650). Geographic region also played a significant role in dual use prevalence. Compared to adults in the Northeast, those in the Midwest (OR = 1.308, 95% CI: 1.137–1.504, p < 0.001), South (OR = 1.420, 95% CI: 1.253–1.609, p < 0.001), and West (OR = 1.606, 95% CI: 1.338–1.929, p < 0.001) had significantly higher odds of dual use. Education was a significant predictor, with higher education levels generally associated with greater odds of dual use. Adults with some college education or an associate degree had higher odds of dual use compared to those with less than a high school education (OR = 1.357, 95% CI: 1.149–1.603, p < 0.001). Similarly, those with a bachelor’s degree had elevated odds of dual use (OR = 1.343, 95% CI: 1.068–1.688, p = 0.012). However, having an advanced degree did not significantly alter the likelihood of dual use compared to the reference group (OR = 1.170, 95% CI: 0.911–1.504, p = 0.217). Poverty status was not a significant predictor of dual use (OR = 0.970, 95% CI: 0.860–1.095, p = 0.622). Similarly, health insurance status did not significantly influence dual use, with odds ratios close to 1 across different insurance categories, indicating no substantial difference in dual use based on insurance type.

The results presented in Table 3 highlight the sociodemographic predictors of dual use of e-cigarettes and combustible cigarettes in the general adult population. Age was a significant predictor of dual use among adults. Compared to the reference group of 18 to 24 years old, adults aged 25 to 34 years old showed slightly higher odds of dual use, although this was not statistically significant (OR = 1.074, 95% CI: 0.935–1.234, p = 0.308). However, as age increased, the likelihood of dual use decreased significantly. Adults aged 45 to 54 years had significantly lower odds of dual use (OR = 0.683, 95% CI: 0.588–0.794, p < 0.001), and this trend continued with even lower odds for older age groups, with the lowest odds observed in those 75 years or older (OR = 0.020, 95% CI: 0.010–0.043, p < 0.001). Male sex was associated with higher odds of dual use compared to females (OR = 1.185, 95% CI: 1.069–1.314, p = 0.002), indicating that males in the general population are more likely to engage in dual use of e-cigarettes and combustible cigarettes. Hispanic ethnicity was strongly associated with a lower likelihood of dual use (OR = 0.479, 95% CI: 0.409–0.560, p < 0.001). When examining race, White individuals had significantly lower odds of dual use compared to other racial groups (OR = 0.542, 95% CI: 0.452–0.651, p < 0.001), whereas there was no significant difference in dual use among Black individuals compared to the reference group (OR = 0.935, 95% CI: 0.790–1.107, p = 0.431). Geographic region was also a significant predictor. Compared to adults in the Northeast, those in the Midwest (OR = 1.320, 95% CI: 1.139–1.531, p < 0.001), South (OR = 1.367, 95% CI: 1.203–1.554, p = 0.000), and West (OR = 1.217, 95% CI: 1.025–1.445, p = 0.025) had higher odds of dual use, suggesting regional differences in dual use patterns. Education level showed a complex relationship with dual use. Adults with a GED had significantly higher odds of dual use compared to those with less than a high school education (OR = 1.362, 95% CI: 1.147–1.618, p = 0.001). However, higher levels of education, such as a bachelor’s degree (OR = 0.406, 95% CI: 0.323–0.510, p < 0.001) and advanced degrees (OR = 0.221, 95% CI: 0.171–0.287, p < 0.001), were associated with significantly lower odds of dual use, indicating that higher educational attainment may be protective against dual use in the general population. Poverty status was associated with higher odds of dual use (OR = 1.195, 95% CI: 1.053–1.357, p = 0.006), suggesting that individuals in poverty are more likely to engage in dual use compared to those above the poverty line. Health insurance type was also a significant predictor. Individuals with any form of insurance, including private insurance (OR = 1.871, 95% CI: 1.552–2.254, p < 0.001), private insurance with some Medicare (OR = 1.823, 95% CI: 1.538–2.161, p < 0.001), and other insurance (OR = 1.584, 95% CI: 1.416–1.773, p < 0.001), were more likely to be dual users compared to those without insurance.

## 4. Discussion

The primary aim of this study was to identify the sociodemographic predictors of dual use of e-cigarettes and combustible cigarettes among U.S. adults using baseline data from the Population Assessment of Tobacco and Health (PATH) Study. By examining key factors such as age, gender, race/ethnicity, and education level, this study sought to provide insights into the characteristics of individuals who are more likely to engage in dual use, which poses significant public health concerns.

Our findings indicate that dual use of e-cigarettes and combustible cigarettes is predominantly observed among younger adults, females, non-Latino Whites, and individuals with higher educational attainment. These results highlight distinct sociodemographic patterns in dual use that differ from traditional smoking behaviors, where lower education and minority status are often associated with higher smoking prevalence.

Previous studies on tobacco use have shown that younger adults are more likely to experiment with e-cigarettes, often perceiving them as less harmful than combustible cigarettes [18–21]. Gender differences in tobacco use have also been noted, with males increasingly engaging in e-cigarette use compared to females but females being targeted [22,23]. The association of dual use with higher education contradicts the traditional inverse relationship between education level and smoking [24]. This pattern aligns with emerging trends where e-cigarettes are marketed as novel, tech-savvy alternatives, potentially appealing to more educated and high socioeconomic individuals [25,26]. Furthermore, racial/ethnic differences in tobacco use are well documented, with non-Latino Whites showing a distinct propensity for electronic cigarette use, possibly reflecting broader cultural and marketing influences.

Several mechanisms may underlie the observed associations. Younger adults might be more prone to dual use due to increased openness to novel products, social influences, and targeted marketing strategies by the tobacco industry. Females’ higher dual use rates could be influenced by different psychosocial factors, such as stress coping mechanisms or perceptions of e-cigarettes as weight control aids. The unexpected association with higher education might reflect a combination of increased awareness of harm reduction strategies and the social acceptability of e-cigarettes in certain demographic groups. The predominance of dual use among non-Latino Whites may be partly due to differential access to and marketing of e-cigarettes, as well as varying cultural attitudes toward tobacco use across racial/ethnic groups.

### 4.1. Limitations

This study has a few limitations. The cross-sectional nature of our analysis limits our ability to infer causality between sociodemographic factors and dual use behaviors. Self-reported data on tobacco use may be subject to recall or social desirability bias. Additionally, the study did not explore behavioral or psychological factors, such as nicotine dependence levels or motivations for use, which could provide further insight into dual use patterns. In addition, the baseline data from the PATH study used in the current analysis were collected approximately 10 years ago. This may reduce the generalizability of the findings to the current US population. Finally, the generalizability of findings may be limited by changes in tobacco product use trends over time since the baseline data collection.

### 4.2. Future Research

Future research should focus on longitudinal analyses to better understand the causal pathways leading to dual use and the transition between e-cigarette and combustible cigarette use over time. Investigating behavioral and psychological factors, such as nicotine dependence, motivations, and perceptions of harm, could elucidate the underlying drivers of dual use. Additionally, exploring the role of socioeconomic status, geographic location, and policy environments may provide a more comprehensive understanding of dual use across diverse populations.

### 4.3. Implications

The findings of this study have important implications for public health interventions and tobacco control policies. Targeted efforts are needed to address the high-risk groups identified, particularly young, highly educated, non-Latino White females. Public health messaging should emphasize the risks associated with dual use and challenge misconceptions about the safety of e-cigarettes. Tobacco control strategies should also consider the influence of marketing and social norms on dual use behaviors, particularly among younger adults and educated populations. Policies that regulate e-cigarette advertising and access, along with robust cessation support tailored to dual users, are critical to reducing the prevalence of this harmful behavior.

## 5. Conclusion

This study provides valuable insights into the sociodemographic predictors of dual use of e-cigarettes and combustible cigarettes among U.S. adults. By identifying the characteristics of high-risk groups, these findings underscore the need for targeted public health strategies to address the unique challenges of dual use. Continued research is essential to further elucidate the mechanisms driving dual use and to develop effective interventions that reduce the burden of tobacco-related harm in the population.

## Figures and Tables

**Table 1.** Descriptive Data (Weighted in Current Smokers)

	%	SE	95%	CI
Age				
18 to 24 years old	15.494	0.004	14.742	16.278
25 to 34 years old	24.082	0.005	23.186	25.000
35 to 44 years old	18.947	0.004	18.107	19.816
45 to 54 years old	19.459	0.004	18.677	20.266
55 to 64 years old	15.077	0.004	14.288	15.901
65 to 74 years old	5.486	0.003	5.007	6.009
75 years old or older	1.455	0.001	1.208	1.752
Sex				
Female	44.231	0.005	43.314	45.153
Male	55.769	0.005	54.847	56.686
Ethnicity				
Non-Hispanic	86.744	0.009	84.920	88.377
Hispanic	13.256	0.009	11.623	15.080
Race				
White	75.109	0.014	72.193	77.812
Black	16.334	0.013	13.939	19.050
Other	8.557	0.005	7.578	9.650
Census Region				
Northeast	16.542	0.012	14.344	19.001
Midwest	24.021	0.013	21.615	26.605
South	40.503	0.013	37.916	43.144
West	18.934	0.010	16.960	21.080
Education				
Less than high school	16.795	0.005	15.920	17.708
GED	10.684	0.003	10.015	11.393
High school graduate	28.548	0.006	27.443	29.678
Some college (no degree) or associate	32.784	0.005	31.713	33.873
Bachelor’s degree or	8.580	0.004	7.810	9.418
Advanced degree	2.609	0.002	2.277	2.988
Poverty				
No	60.430	0.007	58.957	61.884
Yes	39.570	0.007	38.117	41.043
Health Insurance				
Some private insurance	49.077	0.007	47.738	50.418
Private insurance, some Medicare	8.905	0.004	8.234	9.625
Private insurance or Medicare	13.011	0.005	12.112	13.966
Other insurance only	3.446	0.002	3.089	3.842
No insurance	25.560	0.005	24.552	26.596
Dual Use				
No	79.233	0.005	78.312	80.124
Yes	20.767	0.005	19.876	21.688

**Table 2.** Predictors of dual use among current smokers (daily use or some days)

	Odds ratio	Linearized std. err.	95% conf.	interval	p
Age					
18 to 24 years old	Ref.				
25 to 34 years old	0.853	0.063	0.736	0.989	0.035
35 to 44 years old	0.787	0.060	0.677	0.914	0.002
45 to 54 years old	0.647	0.050	0.554	0.755	< 0.001
55 to 64 years old	0.578	0.046	0.493	0.678	< 0.001
65 to 74 years old	0.462	0.069	0.344	0.620	< 0.001
75 years old or older	0.091	0.037	0.041	0.203	< 0.001
Male Sex	0.894	0.042	0.814	0.982	0.019
Hispanic Ethnicity	0.849	0.060	0.738	0.976	0.022
Race					
White	Ref				
Black	0.565	0.052	0.471	0.677	< 0.001
Other	1.041	0.092	0.874	1.239	0.650
Census Region					
Northeast	Ref				
Midwest	1.308	0.092	1.137	1.504	< 0.001
South	1.420	0.090	1.253	1.609	< 0.001
West	1.606	0.148	1.338	1.929	< 0.001
Education Level					
Less than high school	Ref				
GED	1.171	0.108	0.976	1.406	0.088
High school graduate	1.062	0.089	0.899	1.255	0.474
Some college (no degree) or associate	1.357	0.114	1.149	1.603	< 0.001
Bachelor’s degree or	1.343	0.155	1.068	1.688	0.012
Advanced degree	1.170	0.148	0.911	1.504	0.217
Poverty	0.970	0.059	0.860	1.095	0.622
Health Insurance					
Some private insurance	Ref.				
Private insurance, some Medicare	0.973	0.085	0.818	1.157	0.755
Private insurance or Medicare	1.010	0.081	0.861	1.184	0.904
Other insurance only	1.184	0.140	0.937	1.497	0.156
No insurance	0.901	0.054	0.800	1.014	0.084

**Table 3.** Predictors of dual use among general population of adult

	Odds ratio	Linearized std. err.	95% conf.	interval	p
Age					
18 to 24 years old	Ref.				
25 to 34 years old	1.074	0.075	0.935	1.234	0.308
35 to 44 years old	0.917	0.072	0.785	1.071	0.270
45 to 54 years old	0.683	0.052	0.588	0.794	< 0.001
55 to 64 years old	0.527	0.043	0.448	0.620	< 0.001
65 to 74 years old	0.226	0.033	0.170	0.302	< 0.001
75 years old or older	0.020	0.008	0.010	0.043	< 0.001
Male Sex	1.185	0.062	1.069	1.314	0.002
Hispanic Ethnicity	0.479	0.038	0.409	0.560	< 0.001
Race					
White	Ref.				
Black	0.542	0.050	0.452	0.651	< 0.001
Other	0.935	0.079	0.790	1.107	0.431
Census Region					
Northeast	Ref.				
Midwest	1.320	0.098	1.139	1.531	< 0.001
South	1.367	0.088	1.203	1.554	0.000
West	1.217	0.105	1.025	1.445	0.025
Education Level					
Less than high school	Ref.				
GED	1.362	0.118	1.147	1.618	0.001
High school graduate	0.832	0.067	0.709	0.977	0.025
Some college (no degree) or associate	0.873	0.073	0.740	1.029	0.105
Bachelor’s degree or	0.406	0.047	0.323	0.510	< 0.001
Advanced degree	0.221	0.029	0.171	0.287	< 0.001
Poverty	1.195	0.076	1.053	1.357	0.006
Health Insurance					
Some private insurance	Ref.				
Private insurance, some Medicare	1.871	0.176	1.552	2.254	< 0.001
Private insurance or Medicare, s	1.823	0.156	1.538	2.161	< 0.001
Other insurance only	1.744	0.190	1.405	2.166	< 0.001
No insurance	1.584	0.090	1.416	1.773	< 0.001
